# Longitudinal methods for Alzheimer's cognitive status prediction with deep learning

**DOI:** 10.1002/alz.70488

**Published:** 2025-09-25

**Authors:** Houjun Liu, Alyssa Mae Weakley, Hiroko H. Dodge, Xin Liu

**Affiliations:** ^1^ Computer Science Stanford University Stanford California USA; ^2^ Neurology UC Davis Sacramento California USA; ^3^ Neurology Massachusetts General Hospital Harvard Medical School Boston Massachusetts USA; ^4^ Computer Science UC Davis Davis California USA

**Keywords:** Alzheimer's dementia, Alzheimer's Disease Research Centers, data science, deep learning, machine learning, mild cognitive impairment, National Alzheimer's Coordinating Center, transformers

## Abstract

**INTRODUCTION:**

Prediction of amnestic mild cognitive impairment (aMCI) and Alzheimer's disease (AD) using machine learning has primarily focused on short‐term predictions spanning 1–3 years. This study aimed to develop a new machine learning technique to extend predictions of cognitive status over 3–10 years from their last visit.

**METHODS:**

We leveraged deep learning to analyze two longitudinal feature sets: (1) neuropsychological data and (2) neuropsychological data with the addition of patient history data. We also introduce two modeling techniques: (1) to separate normalized baseline features and deviations from baseline, and (2) a new linear attention‐based imputation method.

**RESULTS:**

We demonstrate (1) our technique achieves high 1vA accuracy, representing 81.65% for Control, 72.87% for aMCI, and 86.52% for AD on a 3‐ to 10‐year horizon, and (2) the new method is more accurate than previously proposed approaches for this time horizon.

**DISCUSSION:**

This work offers a new set of techniques for big‐data analysis of longitudinal dementia data.

**Highlights:**

- Develops a new method for the prediction using deep learning of longitudinally verified amnestic mild cognitive impairment (aMCI) and Alzheimer's disease (AD) using the National Alzheimer's Coordinating Center NACC) database.
- Demonstrates comparable performance on the 3‐ to 10‐year prediction horizon, which is significantly more challenging to predict than using the previous approach that only used a 1‐ to 3‐year prediction horizon.
- Highlights that even the prediction of verified 3‐ to 10‐year aMCI that eventually leads to AD is still a challenging task.

## 1 BACKGROUND

Early identification and intervention of amnestic mild cognitive impairment (aMCI), a prodrome of Alzheimer's disease (AD), is particularly valuable because progression to AD can be more easily delayed or arrested at the aMCI stage.^1^, ^2^ This task is complex because at‐risk patients (e.g., those with medical risk factors) who are cognitively normal may either develop aMCI or stay cognitively normal, potentially requiring no intervention. Accurate prediction of individuals who are likely to progress to future stages of AD is a critical task for early intervention implementation. Evidence suggests that AD pathology begins years prior to aMCI symptom presentation,^3^ so the robust prediction of future aMCI and AD onset will be critical for intervention of the condition before clinical signs are apparent.

Unsurprisingly, as the prediction horizon increases, the task of prediction becomes increasingly noisy because the data become more scant and variable. As such, prior work has focused mostly on short‐term prediction over a 1‐ to 3 year horizon^4^, ^5^, ^6^, ^7^ or simply imputing future scores as a proxy for diagnosis, such as the Mini‐Mental State Examination (MMSE).^8^ Of those, only a few works in the prior art attempt to predict future development of aMCI.^5^, ^6^, ^7^ The majority of work was limited to predicting AD, due to the difficulty of predicting aMCI.

**RESEARCH IN CONTEXT**

1. **Systematic review**: We reviewed the literature using traditional sources (e.g., PubMed, Google Scholar, DBLP, etc.) and meeting abstracts in both computer science and neurology. There has been recent interest in longitudinal approaches for amnestic mild cognitive impairment (aMCI) prediction, and the National Alzheimer's Coordinating Center (NACC) database offers a promising venue with which to do so.
2. **Interpretation**: We develop a new deep learning‐based method for the prediction of aMCI and Alzheimer's disease (AD) progression into the 3‐ to 10‐year horizon using the NACC dataset and offer it as a new method of analysis for the community. Since cases of aMCI noted in our work are all verified longitudinally to be prodromal to AD, our work highlights the continued challenge for such predictions.
3. **Future directions**: This manuscript gives a new method for performing the challenging task of 3‐ to 10‐year longitudinal prediction and sets new baselines for future work to iterate upon. Adding new methods such as individualized trajectory prediction or dynamic time warping may provide promising next steps. Furthermore, even if our method is temporal, the reasonably short prediction sequences in our data pipeline mean that long short term memory layer (LSTM) may not be the most computationally efficient solution to the modeling problem. Future work can explore more efficient temporally sequential architectures to improve prediction efficiency.

What does emerge within the prior art, however, is the continued success of big‐data approaches and, in particular, deep learning approaches^9^ for the prediction of AD. One particularly popular machine‐learning method to analyze such time‐series information are deep recurrent neural network (RNN) techniques such as LSTM,^10^ which has shown strong predictive performance for future AD (but not aMCI) prognosis.^4^

In addition to being limited to a 1‐ to 3‐year timespan, prior approaches often relied on summative measures^11^ such as the Geriatric Depression Scale (GDS) and Clinical Dementia Rating (CDR) Dementia Staging Instrument (a system of assesments used to determine dementia state). Reliance on these measures, while engineered to be sensitive to cognitive state, prevents the model from discovering the underlying sources of information that contribute to such scales. In essence, the model simply learns to interpret these scores instead of learning the actual useful underlying features that are more sensitive as markers for the disease. Models trained with this approach, therefore, could be limited in their ability to generalize out of domain and inherit the inductive biases of such summative measures. On the other hand, efforts of manual feature selection^12^, ^13^ are of limited scope, thus preventing the model from identifying new useful measures not previously established in the literature.

Our previous work^6^ demonstrated that transformer models acting on non‐summative data collected at a single‐prior visit achieved reasonable performance (with one‐vs.‐all accuracy at 83% control, 77% aMCI, and 91% AD) on the 1‐ to 3‐year prediction task. Through improved methods in data preparation and architecture, we offer here the first deep‐learning attempt for the prediction of three classes of cognitive status (normal, aMCI, AD) across *3 to 10 years* using data from all prior visits, excluding summative features such as CDR or GDS. Additionally, we simplify the imputation scheme given by our previous work for missing data and introduce a new simplified linear‐attention based imputation scheme that offers a large reduction in parameters.

We have two primary hypotheses. First, we hypothesize that, because all non‐missing values are positive, using a linear projection to enable latent separation of missing values is feasible and performant compared to the previously published transformer imputation method by simply setting missing values to negative. Second, we hypothesize that—due to the long‐term longitudinal performance of the LSTM architecture and the recent evidence that prodromal aMCI occurs many years before symptom presentation^3^—long‐horizon prediction up to 3–10 years for cognitive state (in particular for aMCI) is possible beyond random chance.

## 2 METHODS

### 2.1 Data

#### 2.1.1 Data selection

We follow Liu, et al.^6^ exactly for data selection. Specifically, we leverage the National Alzheimer's Coordinating Center Uniform Data Set Version 3.0 (NACC UDS v3.0) downloaded in June 2022. The NACC UDS is a large longitudinal dataset collected from over 45 National Institutes of Health (NIH) ‐funded Alzheimer's Disease Research Centers (ADRCs) across the United States. This multi‐center dataset includes 45,100 participants, collected from 2006.

We minimally down‐sample a subset of these participants following a few simple criteria to isolate the specific subpopulation we aim to study. In particular, we selected 30,750 participants by filtering based on one of two mutually exclusive criteria that they (1) are labeled as cognitively normal and never develop AD or other types of dementia in the future, that is, they remain cognitively normal for the entire duration of the study (*N *= 19, 743), or (2) are labeled as cognitively normal or aMCI and do develop AD in the future (*N* = 11, 007).

We exclude participants that progress to a non‐AD dementia (e.g., Parkinson's dementia, frontotemporal dementia) from the data set. Furthermore, we filter for participants with at least two evaluation dates (i.e., two samples), with the oldest and most recent sample being at least 3 and no more than 10 years apart (creating the 3‐ to 10‐year criteria). For the results reported at evaluation time, our task uses one sample for each of such spans, ensuring that the desired future timestamp is 3–10 years apart from the last sample collected. At training time, we perform a data augmentation procedure as described in Section 2.1.2 to extract a number of training samples from each of such spans.

We balance the number of samples of patients who did progress from a baseline state with those that did not, resulting in 25,179 patients in total. This step is important because a large amount of the patients do not convert from baseline at all. For instance, a naive prediction always classifying patients as having a cognitive state within normal limits (i.e., “control”) will achieve over 90% accuracy without this procedure. To do this balancing procedure, we determine the class with the smallest number of viable samples (aMCI in this case, *N* = 8393) and perform random uniform drawing without replacement from each class such that our final dataset has exactly 8393 samples per class (making *N* = 25,179).

Naively, after balancing the amount of patients who eventually progress to each state of control, aMCI, and AD, yields very few (i.e., roughly 2000) samples to train our system with. In particular, aMCI created this effect due to the fact that not a lot of patients progressed only to aMCI within the entire sampled period (most stayed within control or eventually moved to AD). Since deep learning approaches benefit from increased scales of data, we describe in Section 2.1.2 an augmentation process to increase the samples with which we can train the system.

#### 2.1.2 Data augmentation

Following this selection process, we then perform an extensive data augmentation process to generate enough samples for longitudinal prediction. In particular, we leverage the fact that, as long as training and evaluation slices are separated by patients, we can reuse each patient's sample multiple times by selecting a subset to predict the exact next sample.

That is, for patient with *t* visits *x*_1_ *, …, x_t_*, instead of just using samples *x*_1_ *, …, x_t−_*_1_ to predict the patient's cognitive state *x_t_*, we create sets:

(1) $$(\left\{x_{i}|i\in 1...j-1\right\},x_{j})|\forall j\in 2...t.$$

**FIGURE 1:**
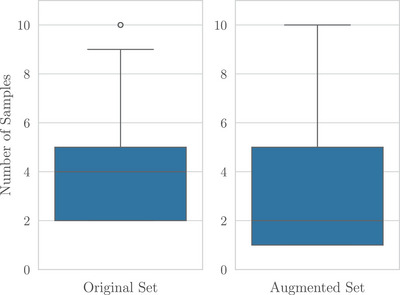
Number of samples in the training set pre‐ and post‐augmentation.

That is, we use each subset of the patients’ visits to predict the next visit beyond that subset. Specifically, we predict a patient's cognitive status at *t*, using all samples that falls prior to *t* at train time and all samples 3–10 years away from *t* at test time. This approach is sound because the LSTM^10^ is a sequence model that is temporally agnostic, so training on more transitions than just the training population will aid in predictive accuracy of any subset's accuracy (such as the 3‐ to 10‐year‐away one). Notably, this augmentation trains the model to make predictions for the 1‐ to 10‐year span (as some subset may not satisfy the 3+ year constraint), and we are only specifically targeting the 3‐ to 10‐year horizon in this work. Hence, the augmented train set is entirely separated from the evaluation set, where this augmentation is *not* applied and where the patients selected do not overlap with those selected in the train set. Figure 1 presents the distribution of sample counts before and after this augmentation.

Although this method introduces a distribution difference between train and test time, the augmented data yield a strict superset of the data that would have been used at test time. As such, it enables more data to be used during training while maintaining distributions similar to those that the model is intended to use at test time.

After all augmentations, we obtain the sample statistics given in Table 1.

**TABLE 1:** Demographic characteristics of the augmented dataset, grouped by final status.

Parameter	Control	Conversions to aMCI	Conversions to AD
N	48,926	8393	23,247
*Age (years)*			
Initial	73.70 ± 0.06	76.90 ± 0.15	77.09 ± 0.09
Final	76.84 ± 0.06	79.47 ± 0.16	79.48 ± 0.09
Follow‐up duration	3.14 ± 0.03	2.58 ± 0.06	2.38 ± 0.03
Education (years)	16.19 ± 0.05	15.98 ± 0.12	15.16 ± 0.09
*Sex*			
Female	31,849	4371	12,589
Male	17,077	4022	10,658
*Race*			
White	40,236	6974	19,595
African American	7121	1084	2426
Asian	1076	233	488
Native American	193	1	25
Pacific Islander	20	26	129
Other	197	21	70
Unknown	83	54	514
*Ethnicity*			
Not Hispanic/Latino	46,399	7910	21,291
Hispanic/Latino	2390	452	1888
Unknown	137	31	68
*Initial status*			
Control	47,777	3561	2098
aMCI	1017^a^	4576	4151
AD	132^a^	256^a^	16,998

^a^Cases who reverted from a more severe cognitive state are not included in analysis. For example, if subjects converted to aMCI then AD between two visits, then our argumentation procedure would yield a sample predicting aMCI for the first timeframe, and AD for the longer timeframe. p95.

Abbreviations: AD, Alzheimer's disease; aMCI, amnestic mild cognitive impairment

### 2.2 Feature selection

As described above, one of our primary aims of this study is to be minimally selective in terms of features, including every available in‐office and non‐invasive marker available in the NACC UDS.^14^

Similar to Liu et al.,^6^ we select two primary groups of features to study: the first group of features is the NACC Neuropsychological Battery (UDS forms C1‐2T)—encompassing broadly global cognitive status, delayed memory, attention, processing, language, visuospatial, and executive function features—which we describe as the “**cognition**” feature set. The second group is data encompassing participant health and prior family history (UDS forms A3–A5)—spanning information regarding familial cognitive impairment history, cognitive risk factors, substance use, psychiatric/mood factors, and so forth—which we call the “**health**” feature set.

Importantly, the prior work^6^ established that the health feature set alone is not a strong correlative factor of AD even cross‐sectionally, and even less so for the 1‐ to 3‐year range prediction of single visits. As such, we do not investigate the use of the health feature set alone and instead created a new dataset combining the “cognition” features with the “health” features for a new “**combined**” feature set, which we benchmark against using the “cognition” data alone.

There are two important considerations to note regarding our feature selection approach. First, the method is minimally selective: we selected essentially every feature available in the NACC UDS regarding these two broad domains of patient history and non‐invasive in‐office markers, allowing the model itself to selectively sample and downweight features as needed. Second, as described above, our method intentionally excludes any summative metrics such as the GDS and CDR, again in order to allow the model to learn the relationships between the observed features instead of simply learning to interpret a summative psychometric measure.

### 2.3 Handling missing data

As with any longitudinal dataset, missing data—both within the feature dimension for a single sample of a visit and systematically for all samples of a given participant—is especially prevalent.

Methods of handling missing data range from interpolation and filling with the mean to more complex neural approaches.^4^, ^5^, ^7^, ^8^ In particular, our previous approach in handling data imputation in the NACC UDS involved using a selective mask on a transformer^15^ attention.^6^ In particular, for each UDS feature *x_k_* represented as a vector in latent space *X_k_ ∈ R^|H|^* (where *H* is the hidden dimension), we project *X_k_* to three vectors *Q_k_*, *K_k_*, and *V_k_* to compute a new hidden vector *X^′^* for each UDS feature via:

(2) $$X\prime_{k}=\sum_{i=0}^{k}\mathrm{softmax}\left(\frac{Q_{j}K_{i}}{\sqrt{d_{k}}}\right)V_{k}\varphi \left(i\right)$$

where the binary masking parameter φ is defined as:

(3) $$\varphi \;\left(i\right)=\left\{\begin{array}{c} 1,\;if\;x_{i}\;exists\\ 0,\;otherwise \end{array}\right.$$

This approach essentially computes a rolling average across all other features modulated by projections of each UDS feature into *Q, K*, and *V;* when a feature is not available, it is masked out by simply being unavailable in this average. Therefore, no matter the amount of feature availability, the output vectors will gather the necessary information to be a useful predictor from the set of features that are available (and, if available, the input vector for that particular feature).

We also explore in this work a simplified version of this imputation scheme, taking advantage of the fact that all non‐missing values are positive. Instead of projecting into three separate vectors, we leverage a simple dot‐product attention^16^: first, we set all missing values to −1; then, we project these values through a single non‐linear function (i.e., two learnable linear maps *S_θ_, T_θ_ ∈ R^H×H^* and an activation function ϕ such that we have *f* (*x*) = *S ·* ϕ *· T* (*x*)); finally, we use these normalized scores as the weights of a weighted average over all samples. That is, for features *x_k_*, we compute:

$$x\prime_{k}=\frac{x_{k}f\left(x_{k}\right)}{\sum_{i=0}^{K}f\left(x_{k}\right)}$$

This linear attention scheme is both more efficient and allows the model to explicitly process the notion of missing values (instead of imputing them away as previous approaches do). Our method enables the recognition of missing values by configuring them to be the only features represented by negative values. We evaluate our approach using both of these imputation methods, reporting the first approach as the “transformers” approach (as they use attention classically used in transformer architecture^15^) and the second, linear attention approach as the “no transformers” approach.

After performing this initial imputation, for *k* UDS features and *t* longitudinal time‐stamps we obtain the input tensor:

$$\left(\begin{array}{ccc} X_{1}^{\left(1\right)} & \text{…} & X_{1}^{\left(t\right)}\\ \vdots & \ddots & \vdots \\ X_{k}^{\left(1\right)} & \text{…} & X_{k}^{\left(t\right)} \end{array}\right)$$

with which we then make a prediction about the patient's cognitive state at time *t*+1 following the procedure given in Section 2.4.

### 2.4 Modeling

In this work, the primary novel contribution involves extending^6^ to model longitudinal data. To do so, we introduce here a new split architecture for modeling such longitudinal data. In particular, our modeling approach is inspired by insights of *intrapersonal variability* as being sensitive for MCI^17^, ^18^: that early instances of MCI may be sensitive to not only changes in cognitive status as compared to cognitively normal individuals, but also changes within a single individual.

Hence, unlike prior approaches, which simply used a single model to directly analyze the longitudinal data^7^, ^8^ or methods that analyzed cognitive state conversion without a notion of sequentiality in longitudinal data,^5^ our modeling approach aims to surface both systematic variability and intra‐personal longitudinal variability as separate input signals explicitly. We now describe this approach in two stages: first, Section 2.4.1 highlights the feature engineering we perform to surface such sequential features; then second, Section 2.4.2 describes our final model architecture.

#### 2.4.1 Feature engineering

In order to surface the difference between inter‐participant changes and intra‐participant cognitive status decline, we perform *two separate normalization steps*.

First, we normalize all input features by computing standardized z‐scores against the cognitively normal population in the training data. In order to prevent data contamination, validation samples are also normalized against the control population.

Second, we novelly normalize all other samples against the first sample by converting them to the ratio against the first sample. That is, suppose feature *j* had value *xj* ^(1)^ at the first sample, and *xj* ^(^ *^t^* ^)^ at time *t*; this step gives $x\prime_{j}^{\left(t\right)}=\frac{x_{j}^{\left(t\right)}}{x_{j}^{\left(1\right)}}$. In essence, since scores are normalized against control already, this step creates features representing the magnitudes of deviation of the patient from their first visit over time.

Notably, two sets of features are created at this point: first, the normalized values against the training control population (the “**baseline**” features); second, the scalars of features against the first visit data for each participant (the “**change**” features).

Since many variables, in particular those involving familial history, do not change across visits, including them in the “change” feature with a constant rate of change of 1 does not add additional information to the model; hence, we mask out (as if the data isn't available) such variables in the “change” set and leave them only in the “baseline” set.

#### 2.4.2 Architecture

Recall that for each feature set, our model requires two sets of inputs: the first analyzing the “baseline” features, and the second input being a time‐series model for analyzing the “change” features.

In order to do this, we employ a late‐fusion approach in constructing our model, presented in detail in Table 2. In particular, we separately encode each group of features. First, we encode the “baseline” features using one of the two imputation methods given in Section 2.3 (quadratic attention with a Transformer or linear attention). Second, we encode the “change” features using an RNN, in particular, a LSTM^10^, initializing the LSTM cell and hidden state with all zeros and returning the final hidden state.

**TABLE 2:** Model topology.

Component	Layer	Specifications
Transformer	Input Proj.	Linear(in: 1, out: 128)
	Transformer encoder	Heads: 4, layers: 3
Temporal encoder	LSTM	Hidden size: 128
		Num layers: 3
Overall model	“Baseline” input	With transformer: (transformer above) without: linear(in: num features, out: hidden)
	“Change” input	(Temporal encoder above)
	Post‐temporal proj.	Linear(in: num features, out: hidden)
	Temporal mix gate (δ)	Linear(in: hidden, out: 1)
	FFNN	δ Temporal + (1 *δ*) Baseline
		Dropout(0.5)
		Linear(in: hidden, out: hidden)
		ReLU
		Linear(hidden, num classes)

Abbreviations: FFNN, feed‐forward neural network; LSTM, long short‐term memory layer.

Then, we project the resulting LSTM hidden state to a scalar gate (δ), which is used to compute a weighted average between the “baseline” encoding and the “change” LSTM encoding. In particular, we compute δ *·* Temporal + (1 − δ) *·* Baseline to determine the full final encoding.

We construct this gate projection using a linear projection without bias. A useful property of such a projection is that, suppose a particular patient had only one sample (and hence no change features), the LSTM hidden state would remain all zeros and therefore the gate value would be 0, naturally ignoring the temporal encoding (i.e., multiplying it by a factor of 0) in cases where there is no change to any of the variables.

Finally, this fused encoding is then processed by a feed‐forward neural network (FFNN) before being projected into a classification head of three classes representing Control, aMCI, and AD dementia. This output is then projected to a 3‐dimensional probability distribution using a softmax activation and finally supervised using standard Cross Entropy loss with mean averaging.

### 2.5 Training and evaluation

Training and deciding the reported final parameters took place in two stages. Prior to all optimization, the dataset was split into 10 folds in a K‐fold fashion. Using these folds, we determined the optimal parameters and then performed K‐fold validation.

Throughout all experiments, the model was trained using a batch size of 64 over 55 epochs of data (or until earlier convergence through early stopping, as measured by dev set accuracy) and using three layers of both the transformer encoder (when active) and feed forward neural network.

First, we conduct a parameter sweep to determine the hyper‐parameters that would maximize dev‐set performance on only the first fold. In particular, we determined that the model variant with quadratic attention performed the best with a hidden size of 512 and a learning rate of 5 *×* 10−5, while the model variant with linear attention performed the best with a hidden size of 2048 and a learning rate of 5 *×* 10−6.

Second, we then collected performance information of the models by training them on all 10 folds and evaluating each on the held‐out fold using the best‐found parameters above. We repeated this across both model attention architectures, as well as both overall feature sets (“cognition” and “combined”). All reported results are single‐value two‐tailed *t*‐test confidence bands for 95% of the results across all 10 folds.

Optimization was performed using the Adam optimizer with weight decay,^19^ with the weight decay parameter set to 0.01. All runs were performed on NVIDIA RTX A5000 GPUs.

## 3 RESULTS

We report our primary results in Table 3. In particular, the table consists of the performance of the model on the validation slices after training using 10‐fold cross‐validation, reporting p95 confidence bands. Our cross‐validation procedure was conducted by partitioning *patients*, not *samples*, into 10 folds, ensuring that there is no leakage of validation information between training and testing. To extract one‐versus‐all accuracy for a specific label from a three‐class classification model, we treat the model predicting that class under test as “positive” and treat any other predicted outcome as “negative.”

**TABLE 3:** Comparison of model performance across architecture choices and featuresets.

	LSTM + linear attn.		LSTM + transformer	
Metric	Combined	Cognition	Combined	Cognition
Overall accuracy	**70.53 ± 1.12**	68.93 ± 0.65	63.76 ± 0.83	68.00 ± 0.81
One‐versus‐rest AUC	**86.01 ± 0.72**	83.91 ± 0.49	80.98 ± 0.57	83.62 ± 0.66
*Control*				
Precision	**70.88 ± 1.32**	70.28 ± 1.43	64.98 ± 3.67	70.76 ± 1.62
Recall	**76.52 ± 1.26**	72.09 ± 1.73	65.27 ± 4.15	68.62 ± 3.08
One‐versus‐rest Acc.	**81.68 ± 0.82**	80.52 ± 0.86	76.35 ± 1.17	80.03 ± 0.67
*aMCI*				
Precision	**59.54 ± 1.40**	58.56 ± 0.90	53.06 ± 1.50	57.26 ± 1.37
Recall	58.00 ± 2.21	58.66 ± 1.62	55.47 ± 6.27	**61.34 ± 2.56**
One‐versus‐rest Acc.	**72.87 ± 0.95**	72.38 ± 0.60	68.65 ± 0.78	71.78 ± 0.76
*AD*				
Precision	**81.52 ± 1.41**	78.26 ± 1.09	75.58 ± 2.09	77.55 ± 1.24
Recall	**77.09 ± 1.76**	76.04 ± 0.79	70.53 ± 1.41	74.05 ± 1.64
One‐versus‐rest Acc.	**86.52 ± 0.69**	84.96 ± 0.57	82.52 ± 0.74	84.19 ± 0.70

*Note*: Two‐tailed *t*‐test across 10‐folds, p95 confidence band reported. Best results per row bolded.

Abbreviations: Acc., accuracy; AD, Alzheimer's disease; aMCI, amnestic mild cognitive impairment; AUC, area under the curve; LSTM, long short‐term memory layer.

**Including health data improves performance**. Including the participant health information resulted in the strongest results across all metrics except (statistically insignificantly) aMCI one‐versus‐rest recall when modeled using the linear attention imputation scheme. Notably, the transformer encoder imputation approach did *not* benefit from including participant health information.

**Linear attention performs slightly better than the transformer encoder**. Across all summative features (overall accuracy and one‐vs.‐rest area under the cureve [AUC]); the linear attention imputation scheme performed better (*p* < 0.05) compared to the quadratic transformer encoder imputation approach. This effect, however, is diminished when measuring the precision and recall of individual classes, with the performance difference between two approaches being statistically insignificant (*p* > 0.05), and in fact with the mean being flipped

**FIGURE 2 alz70488-fig-0002:**
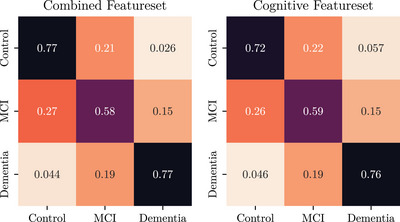
Confusion matrix of model performance across both feature sets using linear attention.

**FIGURE 3 alz70488-fig-0003:**
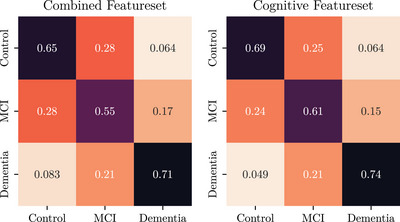
Confusion matrix of model performance across both feature sets using transformer encoder.

**Predicting aMCI remains a challenge**. In the error analysis presented in the confusion matrices given in Figures 2 and 3, we note that the vast majority of errors (across both approaches) are centered around confusion between Control with aMCI and confusion between aMCI and AD dementia, that is, even in this *3‐ to 10‐year horizon* case, prediction of future incidence of progression into AD dementia is much less challenging than future incidence of aMCI.

## 4 DISCUSSION

In this work, using longitudinal data from the NACC UDS (spanning 2–10 years) as input features, we introduce a set of new models and techniques to predict a participant's cognitive state in 3–10 years from their last visit. We introduce[Fig alz70488-fig-0002], [Fig alz70488-fig-0003] four primary contributions. First, we give competitive results on the new long time horizon prediction task to previously presented shorter predictions, in particular doing so *without* using any summative features such as the CDR, but the exam features which correlates to the CDR, such as the MMSE, were used. Second, we introduce a new linear‐attention based formulation for handling missing data, which we show to be more effective than our previous state‐of‐the‐art^6^ transformer‐based imputation scheme. Third, building on previous literature regarding *intrapersonal variability* as being a sensitive measure for the development of aMCI,^17^, ^18^ we introduce a new feature engineering scheme that separates invariant “baseline” features from the variant “change” features, allowing the models to analyze both jointly as separate input feature sets. Lastly, in this study, we controlled carefully for our label of aMCI to reduce the likelihood that individuals would progress to a non‐AD type of dementia. Due to the nature of longitudinal data available in the NACC UDS, we were able to confirm that each patient we labeled as aMCI indeed progressed into AD dementia in the future. Yet, prediction accuracy still suffers with this enhanced label.

The results given by our work confirm both of our hypotheses. First, for Control and AD prediction, the lightweight linear imputation scheme shows statistically significant improvement for one‐versus‐all accuracy, and the method yields no statistically significant detriment over the previously published Transformer imputation scheme while being more parameter efficient. Second, we show comparable performance in the 3‐ to 10‐year prediction horizon task compared to previous results that span only a 1‐ to 3‐year horizon. In particular, we demonstrate 81.65% control, 72.87% aMCI, and 86.52% AD one‐versus‐all accuracy on the prediction of patient cognitive state 3–10 years from the baseline. Despite the longer predictive horizon, this result is comparable in accuracy to previously reported approaches for the cognitive status prediction task in the much shorter 1‐ to 3‐year horizon: 83% control, 77% aMCI, and 91% AD in Liu, 2024,^6^ 74.5% for aMCI conversion in Lin and 2018.^5^ This result, in particular, for not using any invasive measures such as cerebrospinal fluid (CSF) or magnetic resonance imaging (MRI) values, represents a significant elongation of the possible horizon in which early intervention may be possible.

One notable finding regarding our modeling technique involves the fact that while combining participant history (UDS forms A3–A5) and cognitive status (UDS forms C1‐2T) for joint analysis yielded the highest overall results, our previous Transformer encoder imputation approach^6^ did not benefit from including the patient history information. One possible interpretation of this involves the fact that there exists little variance within the patient history information, meaning the transformer‐based model was not able to learn much present relationships between features for imputation as intended when the approach was proposed.^6^ As such, a simpler scheme of linear attention, which simply attended to the representation of there being a “feature missing,” performed equally well on many metrics and better on the overall metrics. Yet, it is important to emphasize that, given the novel improved modeling presented in this work, the longitudinal setting does benefit from the inclusion of patient health history information in addition to cognitive information, creating an avenue for future study.

This work further reveals that, though neural metrics are becoming increasingly competitive at the prediction of control and AD at even long time‐frames, the prediction of aMCI remains a challenge. Recent work leveraging individualized trajectory prediction^20^ using techniques such as dynamic time warping^21^ may present a compelling avenue for future analysis. Furthermore, due to the relatively short size of input sequences, LSTMs may not be the most computationally efficient solution to the modeling problem. Future work can explore the use of more efficient, temporally sequential architectures to improve prediction efficiency, such as flow matching.

Overall, we present here a new set of techniques and competitive results for the 3‐ to 10‐year horizon prediction of control, aMCI, and AD incidence without using summative metrics. The methods developed here may be illustrative for future longitudinal analysis of the disease using big‐data techniques.

## CONFLICT OF INTEREST STATEMENT

The authors have no conflicts of interest to declare for this work. The NACC database used in the work is funded by NIA/NIH Grant U24 AG072122.Author disclosures are available in the Supporting Information.

## INFORMED CONSENT

We did not process human subject data beyond those already collected in the NACC database referenced above. Separate informed consent for this study, therefore, was not necessary.

## Supporting information



Supporting Information
